# Compound Ex Vivo and In Silico Method for Hemodynamic Analysis of Stented Arteries

**DOI:** 10.1371/journal.pone.0058147

**Published:** 2013-03-13

**Authors:** Farhad Rikhtegar, Fernando Pacheco, Christophe Wyss, Kathryn S. Stok, Heng Ge, Ryan J. Choo, Aldo Ferrari, Dimos Poulikakos, Ralph Müller, Vartan Kurtcuoglu

**Affiliations:** 1 Laboratory of Thermodynamics in Emerging Technologies, Department of Mechanical and Process Engineering, ETH Zurich, Zurich, Switzerland; 2 Department of Bioengineering, Imperial College, London, United Kingdom; 3 Clinic of Cardiology, University Hospital Zurich, Zurich, Switzerland; 4 Institute for Biomechanics, Department Health Sciences and Technology, ETH Zurich, Zurich, Switzerland; 5 The Interface Group, Institute of Physiology, University of Zurich, Zurich, Switzerland; King’s College London School of Medicine, United Kingdom

## Abstract

Hemodynamic factors such as low wall shear stress have been shown to influence endothelial healing and atherogenesis in stent-free vessels. However, in stented vessels, a reliable quantitative analysis of such relations has not been possible due to the lack of a suitable method for the accurate acquisition of blood flow. The objective of this work was to develop a method for the precise reconstruction of hemodynamics and quantification of wall shear stress in stented vessels. We have developed such a method that can be applied to vessels stented in or ex vivo and processed ex vivo. Here we stented the coronary arteries of ex vivo porcine hearts, performed vascular corrosion casting, acquired the vessel geometry using micro-computed tomography and reconstructed blood flow and shear stress using computational fluid dynamics. The method yields accurate local flow information through anatomic fidelity, capturing in detail the stent geometry, arterial tissue prolapse, radial and axial arterial deformation as well as strut malapposition. This novel compound method may serve as a unique tool for spatially resolved analysis of the relationship between hemodynamic factors and vascular biology. It can further be employed to optimize stent design and stenting strategies.

## Introduction

Atherosclerosis is the leading cause of death in most developed countries, predominantly as a result of myocardial infarction due to coronary heart disease (CHD). Percutaneous coronary intervention (PCI) that generally involves the placement of a stent has become the primary mode of CHD treatment over the past 20 years [1].

CHD is characterized by progressive atherosclerotic plaques that narrow (stenose) the coronary artery lumen, thereby reducing blood flow to the myocardium. PCI is used to expand the lumen with a balloon catheter and to keep it open with a wire scaffold (stent).

Despite stent placement, incidence of renewed stenosis of the vessel can be as high as 30% [2], [3], most commonly due to neointimal hyperplasia (NIH) [4]. NIH is linked to both the injury or destruction of the endothelium [5], [6] and the loss of smooth muscle cells (SMC) due to stretching of the intima during stent deployment [7]. Expedient endothelial regeneration reduces NIH [8], [9], and endothelial regeneration itself is influenced by blood flow. Similarly, the distribution of atherosclerotic plaques is strongly influenced by the local wall shear stress (WSS) distribution [10], [11]. As WSS is proportional to the gradient of blood flow velocity at the endothelium, precise knowledge of hemodynamics is necessary to derive it. The required level of precision can currently not be achieved clinically using phase-contrast magnetic resonance imaging (PC-MRI) [12], Doppler ultrasound, or other flow measurement techniques [13−15]. For this reason, flow field reconstruction using computational fluid dynamics (CFD) based on medical image data has become the state-of-the-art for determining WSS in stent-free vessels [11], [16]–[19].

A prerequisite for deriving WSS in stented arteries using CFD is the precise definition of the stent geometry with feature sizes of the order of tens of microns. However, no current clinical imaging modality can yield a three-dimensional (3D) representation of a deployed stent with sufficient accuracy for reliable CFD calculations. Computed tomography (CT) [20, MRI [21], intravascular ultrasound [11], [18], [22] and digital angiography [23] do not offer sufficient spatial resolution to capture individual stent struts in detail, and optical coherence tomography is limited by the opacity of the struts to the emitted light.

To circumvent these limitations, hybrid approaches have been developed where the stent-free artery is acquired via CT, digital angiography or MRI, and a virtual stent is placed in the generated digital dataset prior to the calculation of WSS [24]–[31]. Other methods omit in vivo imaging completely [32]–[39], for example by performing image acquisition on explanted stented arteries using micro-computed tomography (µCT) [32], [33], or by placing stents in artificial artery models and then proceeding with µCT [33]–[39].

The individual methods have their respective strengths and weaknesses. While some optimize processing speed and cost by approximating the deployed stent in a computer aided design (CAD) environment [29], others opt for slower, more expensive but also more accurate approaches based on computational structural mechanics simulations of stent deployment [27]. Further methods give preference to actual rather than virtual stent deployment, thereby sacrificing the flexibility of computational techniques for the possibility to capture the expanded stent geometry with higher fidelity when real arteries are used [32], or for the possibility to investigate complex stenting procedures such as double stenting of main vessel and side branch [35]. Some approaches, finally, do not consider derivation of WSS [40]–[42].

In situations where destructive processing of a stented artery is not an issue, combination of vascular corrosion casting (VCC) with µCT and CFD may yield detailed reconstruction of WSS distribution. VCC, originally developed for producing anatomical specimens, can generate negatives of entire vascular trees with sub-micron accuracy, while µCT can be used to digitize the VCC cast with sufficient resolution to capture stent struts in detail. LaDisa and coworkers were the first to combine these methods by stenting rabbit iliac arteries in vivo, sacrificing the animals after two or three weeks, casting the artery lumen, macerating the surrounding tissue and removing the stent with a sanding pad before acquiring the lumen negative by µCT [43]. However, individual stent struts could not be resolved with their technique.

Here we present a method that combines VCC, µCT and CFD to a platform for the precise calculation of WSS in stented arteries. This method is able to accurately resolve both the macroscopic arrangement of stented vessels as well as the microscopic structure of the stent struts.

## Methods

An expanded methods section is provided in the supporting information online (Appendix S1).

### Heart Preparation and Stenting

Porcine hearts obtained with permission from the local slaughterhouse (Metzegerei Angst AG, Zurich, Switzerland) were cannulated in preparation for stenting of the LCA. Absorbable metal scaffolds of 10 mm length and 3 mm diameter (Biotronik AG, Bülach, Switzerland) were placed by an interventional cardiologist under angiographic guidance using the manufacturer-specified inflation pressure of 12 bar.

### Vascular Corrosion Casting

A 1∶0.1225 by weight mixture of low-shrinkage epoxy-based Biodur E 20 (EP20–EP22) resin (Biodur Products GmbH, Heidelberg, Germany) and iodine-saturated methyl ethyl ketone solvent was used as a radio-opaque casting material [44]. The resin was injected into the stented coronary vascular tree under physiological pressure of 90 mmHg (120 mbar) [45]. After a setting period of 36 hours, the heart was macerated for 12 h at 55°C in a 7.5% w/v solution of potassium hydroxide.

### µCT Imaging of Stented Casts

The stented coronary arteries were first imaged using micro-computed tomography (µCT 80, Scanco Medical AG, Brüttisellen, Switzerland) with an isotropic voxel size of 74 µm (energy 70 kVp, integration time 300 ms, tube current 114 µA, and two times frame averaging) to provide the image data of the overall coronary arterial tree geometry. Following this, the stented sections were removed from the artery tree and re-scanned (µCT 40, Scanco) with an isotropic voxel size of 6 µm (energy 70 kVp, integration time 300 ms, tube current 114 µA, and two times frame averaging) in order to resolve individual stent struts (Figure S1).

### Image Processing

A constrained 3D Gauss filter was used to partly suppress noise in the raw µCT volumes (σ = 1.2, s = 1.0). The coronary artery lumen was segmented from both µCT datasets independently using a semi-automatic, intensity-based approach in Avizo 6.2 (Visualization Sciences Group SAS, Merignac, France). The resulting 3D geometries were registered and merged in Geomagic Studio 12 (Geomagic, Inc., Morrisville, NC, USA) to where the high resolution geometry represented the stented artery region and the lower resolution one the remainder of the arterial tree (Figure S2). The merged geometry was exported in STL format for subsequent computational grid generation.

### CFD Calculations

A computational grid consisting of approximately 48 million tetrahedral elements was generated in the merged geometry in ANSYS ICEM CFD (ANSYS, Inc., Canonsburg, PA, USA); see Figure S3. To calculate flow velocity, pressure and WSS distribution, transient and steady-state computational flow analysis was carried out with the finite volume CFD code ANSYS CFX using a Newtonian fluid model with constant density of 1050 kg/m^3^ and dynamic viscosity of 0.0035 Pa^.^s [46]. Boundary conditions were chosen as follows: No slip at the vessel wall, blood inflow rate of 0.95 mL/s at the ostium for the steady-state calculations [16] and time-dependent flow rate for the transient case according to [17]. Zero relative pressure was set at the outlet with the largest diameter and outflow rates at the remaining outlets were determined according to Murray’s law [47]; see Figure S4. For the transient simulations, two cardiac cycles were calculated using a time step size of 0.01 seconds, but only the data of the second cycle were evaluated to obtain results independent of the initial conditions. With residual reduction to 10^−8^ of the initial value as convergence criterion, the steady-state calculations took approximately two hours on 32 AMD Opteron 6174 processor cores. The transient computations required 25 minutes per time step with a convergence criterion of 10^−6^ at each point in time. Grid independence studies were performed (Appendix S1).

## Results

In the following we will show on ex vivo porcine hearts that the compound method presented herein ensures anatomic fidelity, capturing arterial tissue prolapse, radial and axial arterial deformation as well as stent malapposition. We will further show how this method yields detailed blood flow fields and wall shear stress maps in stented coronary arteries (see Video S1), noting that it can also be used in ex vivo human arteries with minimal change to the protocol.

### Arterial Tissue Prolapse

The commonly used stent-to-artery diameter ratio of 1.1–1.2 [48] in coronary arteries can lead in conjunction with the elasticity of the vessel wall to tissue prolapse [49], [50]. We use the term ‘prolapse’ in accordance with the biomedical engineering literature to refer to any degree of tissue protrusion between the stent struts, noting that in the medical literature it is generally associated with protrusion of plaque or thrombus beyond the inner stent surface. Prolapse affects local hemodynamics, thereby altering WSS. Moreover, tissue prolapse is associated with increased incidence of acute and subacute thrombosis [50].

**Figure 1:**
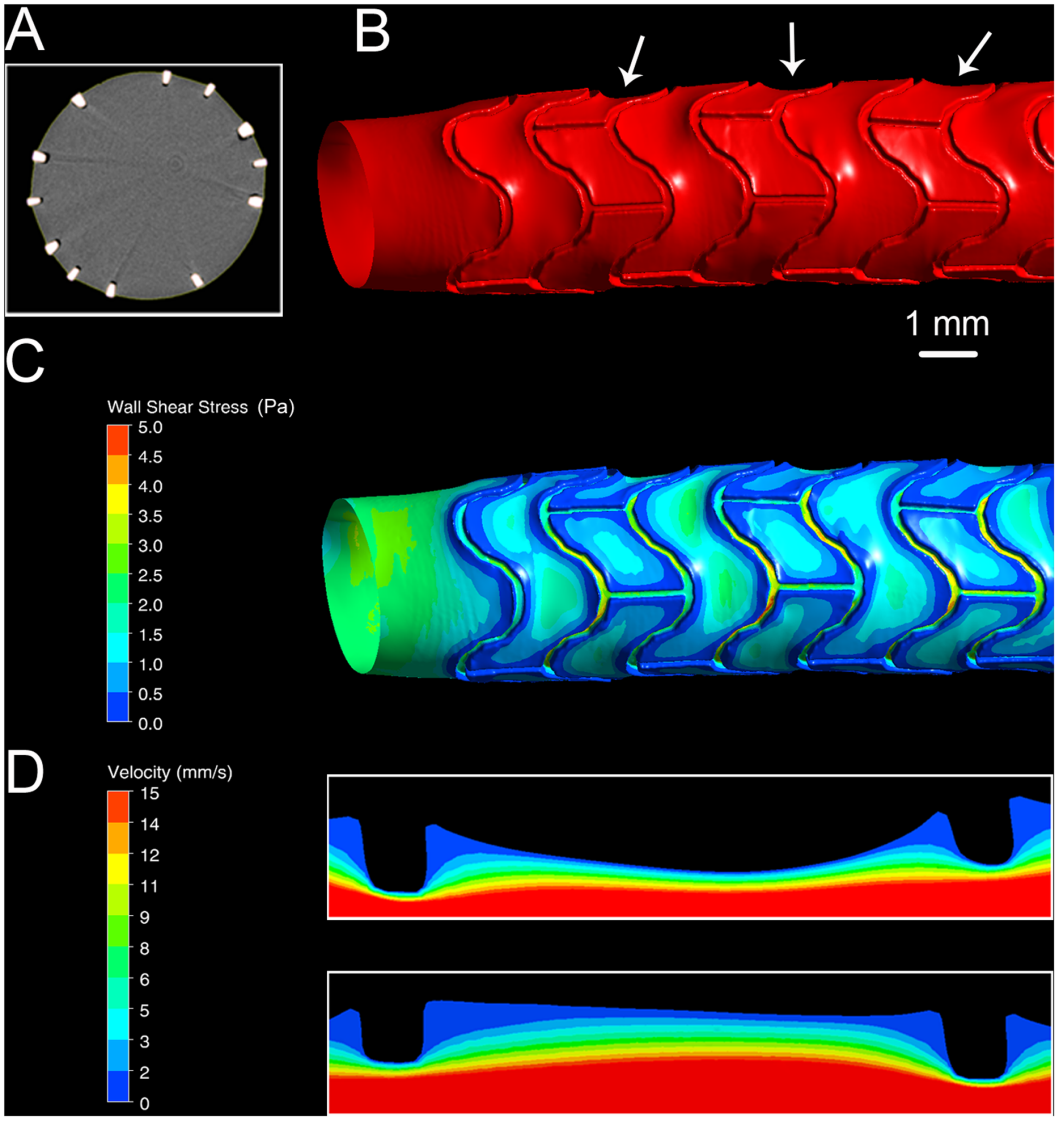
Arterial tissue prolapse between stent struts. (A) Representative micro-computed tomography (µCT) scan section of stented porcine left coronary artery corrosion cast. (B) Reconstructed surface of arterial lumen negative obtained by µCT of a corrosion cast. The white arrows point to prolapsed regions. (C) Wall shear stress (WSS) distribution in the same region shown in B. Higher WSS is evident in prolapsed regions compared to regions without prolapse. Blood flow is from left to right. (D) Velocity contour plots in prolapsed (top) and prolapse-free inter-strut sections. Regions of low velocity are evident near stent struts in both cases, with larger low-velocity regions in the prolapse-free segment. This segment also shows lower near-wall velocity.

As illustrated in Fig. 1, the method captures arterial tissue prolapse. Panel A shows a representative µCT scan section of a stented porcine left coronary artery corrosion cast. Both the cured, contrast-enhanced resin in place of the artery lumen as well as cross-sections of stent struts are clearly visible. The dark areas between resin and struts are due to gas formed in the VCC process through the interaction of stent with resin solution. These areas are merged with the lumen representation in the image segmentation step. Fig. 1B shows the reconstructed surface of a stented porcine left coronary artery (LCA) lumen negative that was acquired using µCT of a corrosion cast. The white arrows between the imprints of the stent struts point to regions of prolapse. These occur most markedly in areas without strut connectors, indicating that arterial tissue prolapse is dependent on stent design.

Fig. 1C shows the corresponding WSS distribution. Higher WSS is evident in regions of prolapse owing to higher velocities near the wall compared to prolapse-free sections as illustrated by the velocity contours in Fig. 1D. Regions of low velocity are recognizable near the struts in both cases, but the size of these regions is clearly different for the two conditions. It is thus evident that arterial tissue prolapse influences hemodynamics and therewith the local WSS distribution with which predictions of plaque development can be made.

### Radial Wall Deformation

**Figure 2:**
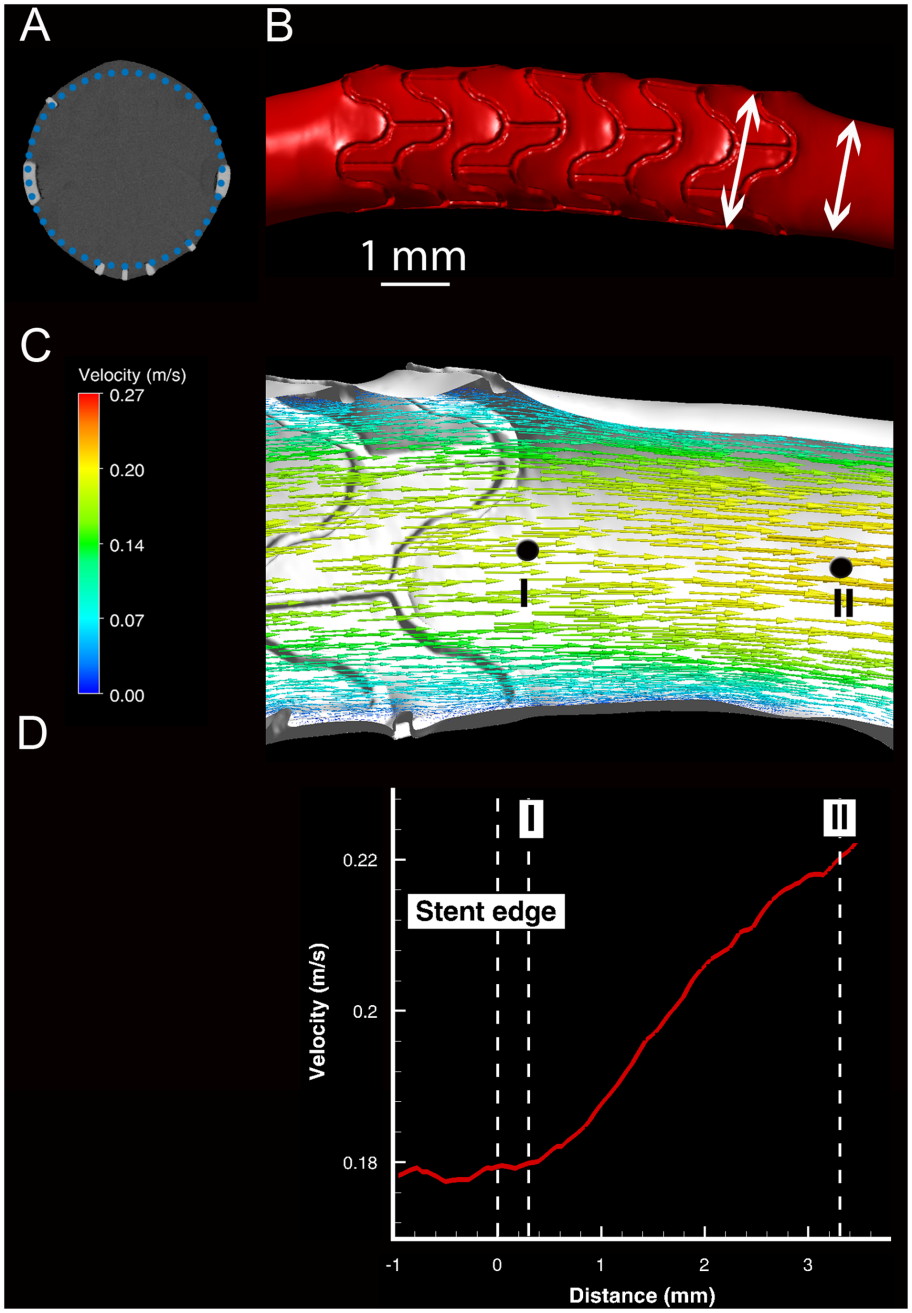
Figure 2. Radial wall deformation of stented artery. (A) Representative micro-computed tomography cross-section of a stented porcine left coronary artery corrosion cast. The dotted circle shows the nominal circular cross-section (B) Radial arterial enlargement caused by stenting. The arrows indicate arterial diameter in the stented (left) and stent-free regions (C) Velocity vectors in the mid-longitudinal section plane of the stented artery. The velocity profile along the vessel centerline from Point I to Point II is shown in (D), where the increase in velocity in the stent-free section is visible. The vertical dashed lines mark the end of the stent and the axial locations of Points I and II shown in panel C.

Histological studies show that stent deployment changes the circular cross-sectional shape of the artery [51]. This method captures such deformations: Fig. 2A depicts a representative cross-section through a stented coronary artery from a µCT image of the lumen. The dotted circle serves as a reference for the local deformation caused by the struts. These deformations impact hemodynamics and WSS. Fig. 2B demonstrates the increase in lumen diameter from the stent-free section of the artery to its stented part, which can lead to a local decrease in both WSS and velocity. This is seen in detail in Fig. 2C: To ensure mass conservation, blood has to accelerate from the stented part of the artery with a larger diameter to the stent-free section with smaller diameter.

### Axial Arterial Deformation

Stenting results in substantial axial deformation, causing a straightening of the artery [52], which affects hemodynamics and WSS distribution substantially.

**Figure 3:**
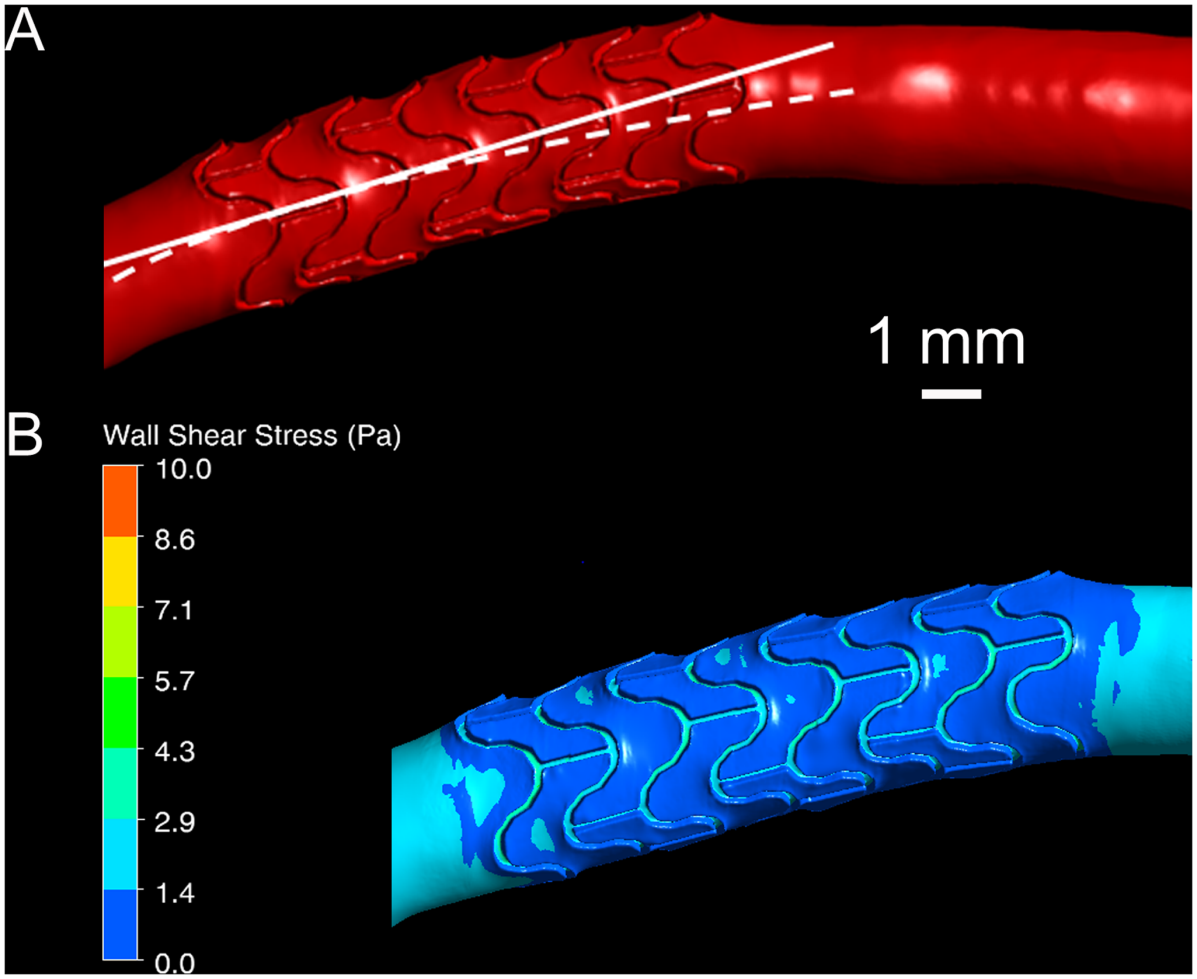
Axial arterial deformation due to stenting. (A) Visualization of arterial centerline change in a stented section. The solid line shows the axis of the stent, while the dashed line approximates the centerline of the stent-free artery. (B) Wall shear stress (WSS) distribution in the same stented artery. An extended area of low WSS is seen immediately downstream of the stent at the outer artery wall due to the change in curvature.

Fig. 3 shows that the herein presented compound method captures axial arterial deformation. The dashed line in Fig. 3A approximates the centerline of the stent-free artery. The solid line illustrates the change of the centerline shape in the stented region. Evaluated from left to right, the sudden straightening of the artery in the stented segment and the abrupt return in curvature to that of the stent-free region are evident. Fig. 3B shows that this leads to an extended area of low WSS at the outer vessel wall. Without the stent, the inner arterial wall would be the main location of low WSS, atherosclerotic plaque formation and neointimal hyperplasia (NIH) [22], [46]. Consequently, axial arterial deformation due to stent placement has to be accounted for in WSS derivations.

### Stent Malapposition

**Figure 4:**
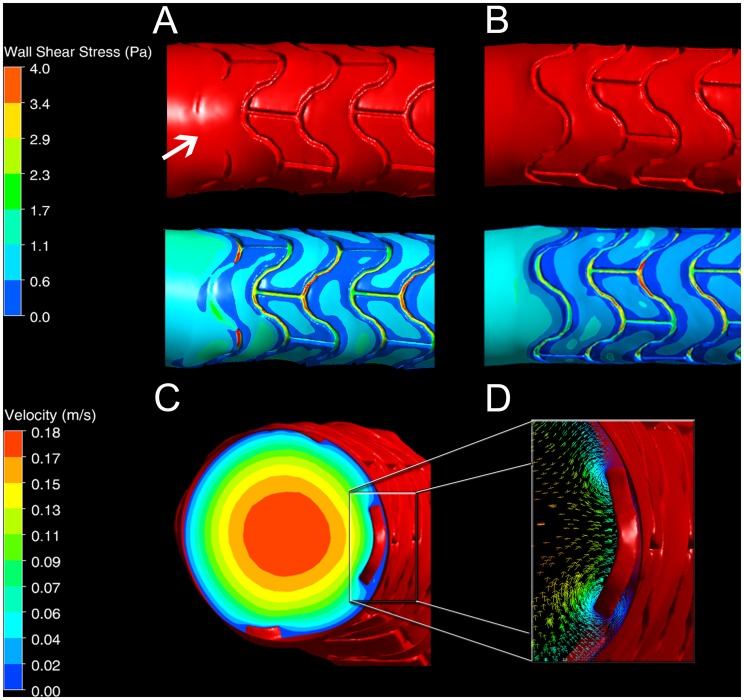
Stent malapposition and its effect on local hemodynamics. (A) Imprint of malapposed stent end section (arrow) in artery lumen negative (top) and corresponding wall shear stress (WSS) distribution (bottom). Higher WSS can be observed in the vicinity of the malapposed strut due to flow tunneling compared to (B), where a similar fully apposed stent end section is shown. (C) Velocity contour in axial cross-section of the stented artery near a malapposed strut. Changes in velocity and division of blood flow can be seen. (D) Velocity vector plot in the vicinity of the malapposed strut demonstrates the presence of vortices. These influence WSS distribution and may lead to thrombosis.

Stent malapposition alters in-stent hemodynamics, causes low WSS distally and is hypothesized to be a major factor in thrombosis [53]. The method presented here captures malapposed struts: Fig. 4A and 4B show the arterial lumen surface and WSS in malapposed and fully apposed stented regions, respectively. The malapposed strut shown in Figs. 4C and 4D causes tunneling of blood flow between the strut and the endothelium, leading to high WSS and perturbation of the local flow field. Such perturbed flow (see Video S2) is associated with increased risk of thrombosis [54].

### Reconstruction of Hemodynamic State

**Figure 5:**
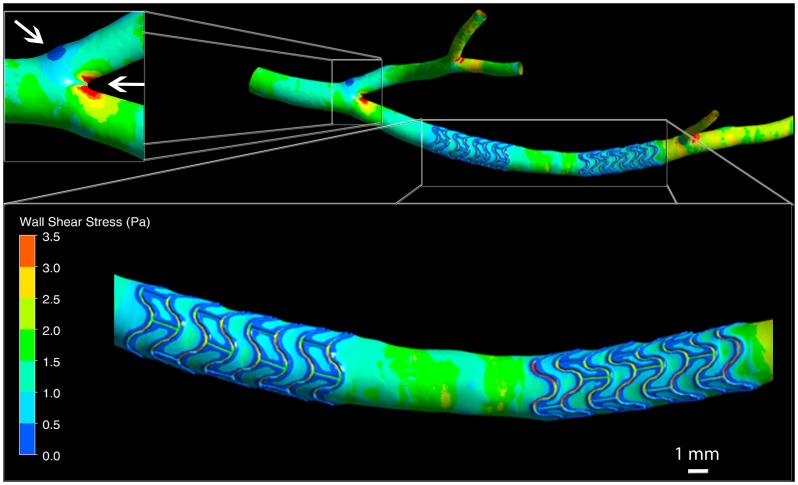
Wall shear stress distribution in a porcine left coronary artery with two stents. The bottom inset shows a magnified view of the stented segments. Wall shear stress (WSS) below 0.5 Pa is reported to correlate with sites of intimal thickening [51]. Such low WSS can be seen here to occur mainly in the vicinity of stent struts and at bifurcations. The left inset shows low and high wall shear stress regions occurring, respectively, at the outer and inner walls of the bifurcation (arrows).

Both the local geometry at the vessel wall, as well as the large scale arterial anatomy, influence WSS distribution and can be accounted for with this method. This can be seen in Fig. 5 where regions of low, atheroprone shear stress are present at bifurcations and nearby individual stent struts. Interestingly, low WSS is not only present adjacent to struts arranged perpendicular to the flow direction, but also occurs in the vicinity of inter-strut connectors arranged parallel to the artery’s longitudinal axis.

**Figure 6:**
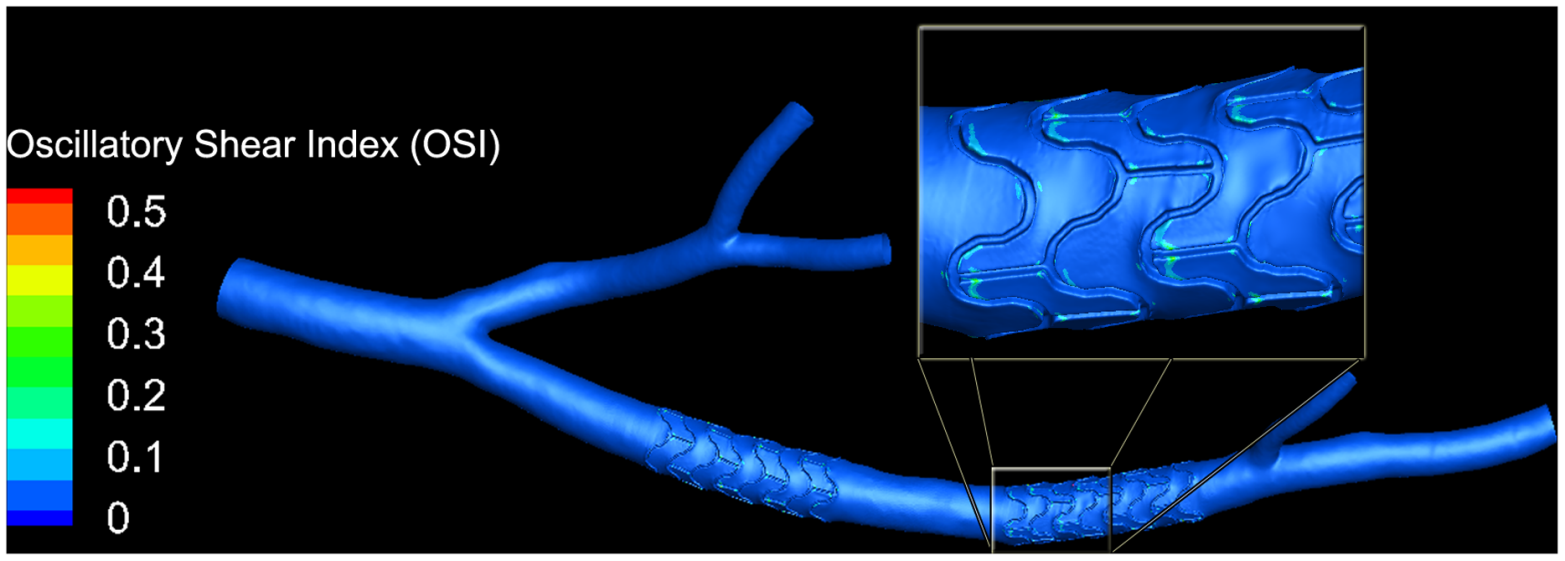
Oscillatory shear index (OSI) distribution in a porcine left coronary artery with two stents. The inset shows a magnified view of part of the second stented segment. Elevated values of OSI have been reported to correlate with atheroprone vessel regions [17]. Areas of increased OSI are visible near strut junctions. They contain small focal spots that reach values close close to the maximum of 0.5.

Figure 6 shows the distribution of oscillatory shear index (OSI), which is a measure of temporal WSS change (see Appendix S1). High values of OSI have been shown to correlate with atheroprone regions of the vessel [17]. Increased values of OSI are seen here near strut junctions, which is in agreement with earlier observations [55].

**Figure 7:**
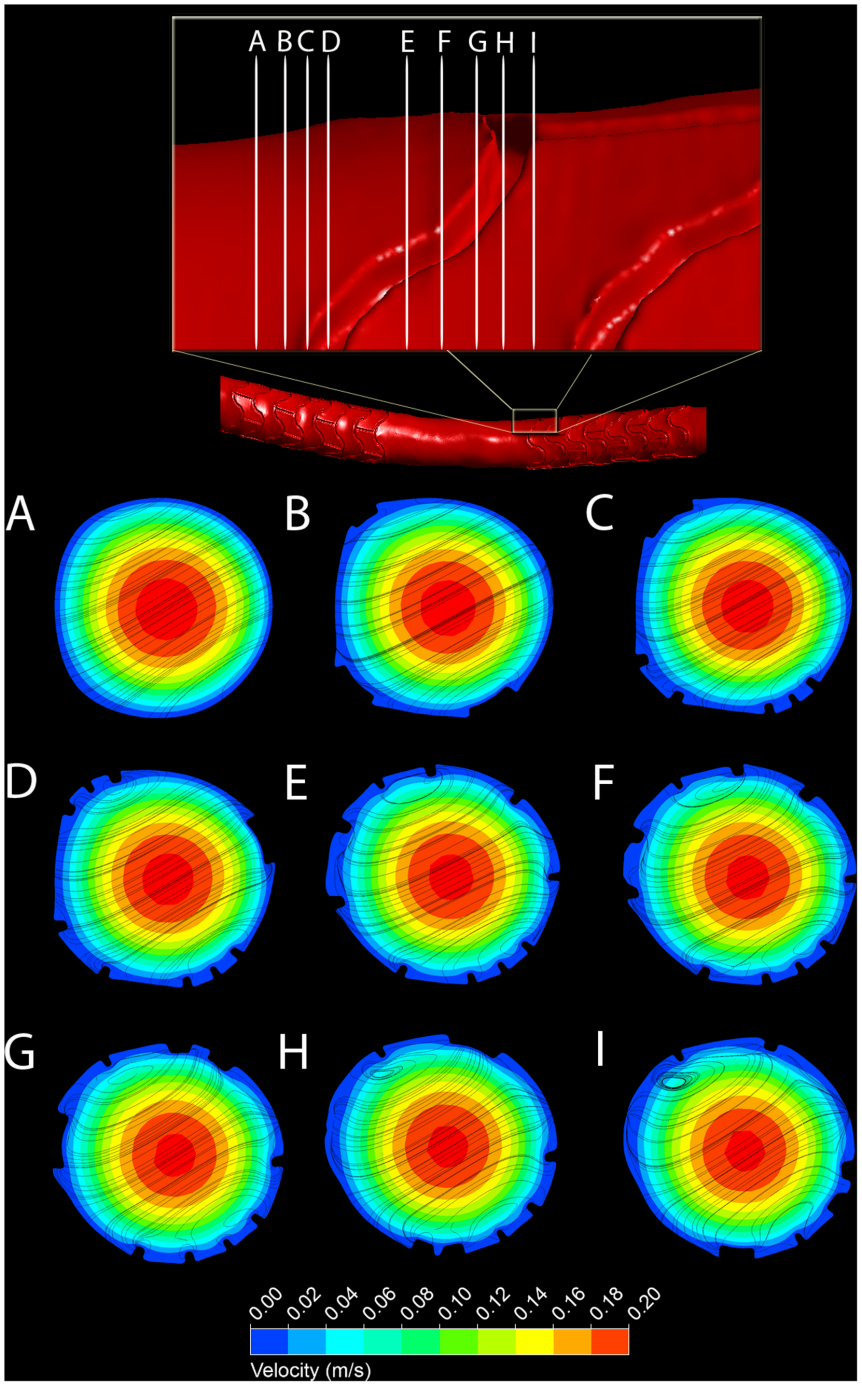
Velocity contours and secondary flow around individual stent struts in a porcine left coronary artery. *Top:* Overview and close-up of reconstructed surface of arterial lumen negative obtained by µCT of a corrosion cast. The labels A to I indicate the location of the cross-sections shown in the bottom panels. *Bottom:* Velocity contour plots at cross-sections A to I. To visualize secondary flow structures, streamlines are derived from velocity vectors projected onto the respective cross-section.

**Figure 8:**
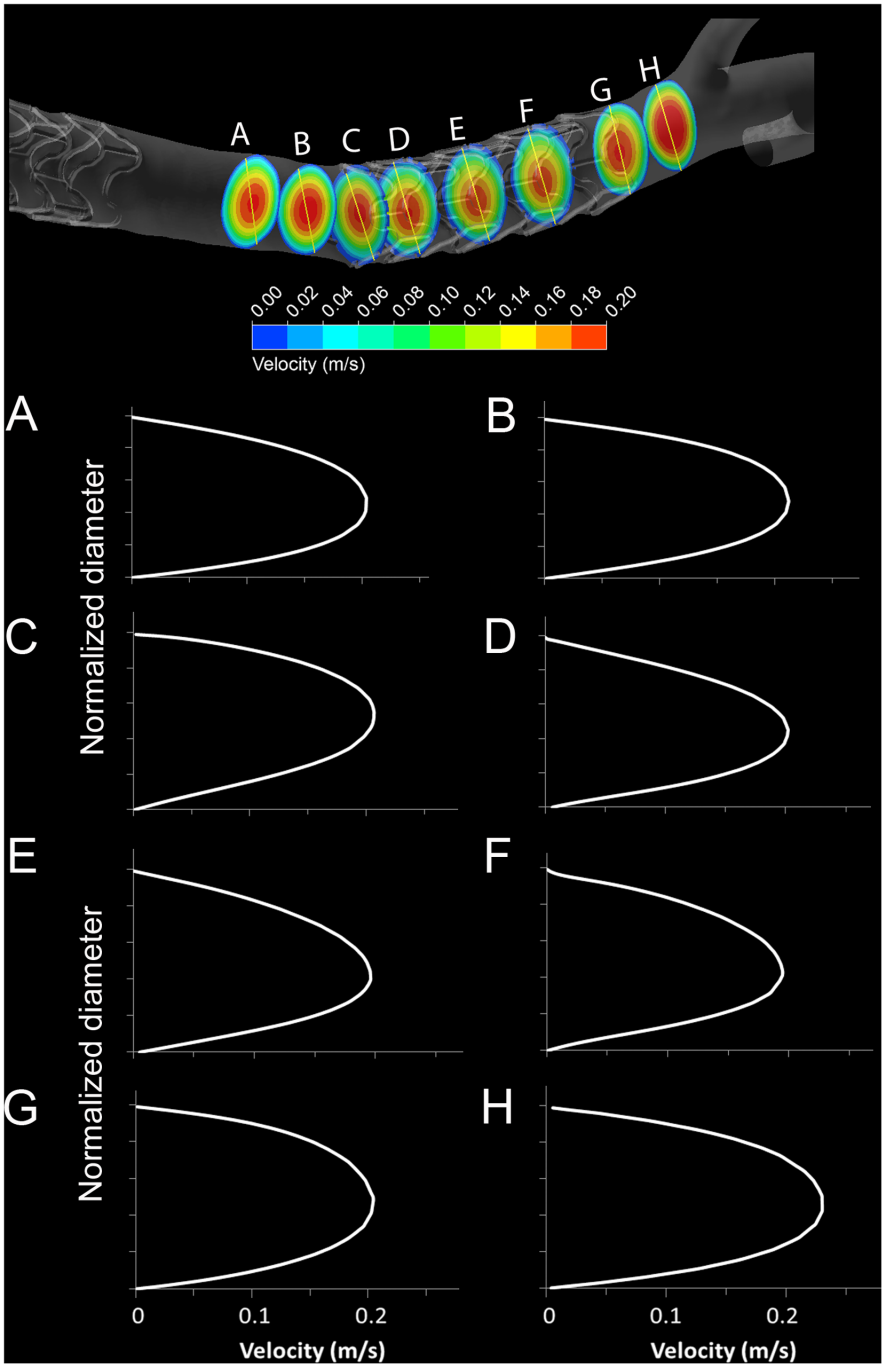
Velocity profiles in a porcine left coronary artery with two stents. Results are shown for cross-sections upstream of the distal stent (A and B), within (C, D E and F) and downstream of the stent (G and H). *Top:* Velocity contour plots. *Bottom:* Velocity projections onto axial planes. The vertical axes are normalized to a common diameter.

The flow structures that lead to low WSS are illustrated in Fig. 7. Panel A shows velocity contours and streamlines projected axially onto a cross-sectional plane immediately upstream of the stent. The velocity profile is near parabolic, and there are no recirculation areas discernible. Entering the stented vessel region in downstream direction (Panel B), flow disturbances begin to develop and quickly lead to the generation of recirculation zones (Panels C to I). In addition, the parabolic velocity profile is altered due to changes in shape of the arterial cross-section, as well as due to the stent struts’ influence on the near-wall flow. This can be seen in the offset of the velocity contours in Fig. 8.

Axial arterial shape change as a result of the stent placement further affect the velocity profile as illustrated in Fig. 8. The top panel shows an artery segment with two stents. Entering the second stent from the upstream direction at cross-section A, the location of peak velocity is deflected from the vessel centerline in plane D due to the increased curvature of the vessel at the stent edge. Effects of the stent strut as described above maintain the off-center profile throughout the length of the stent (planes E and F).

**Figure 9:**
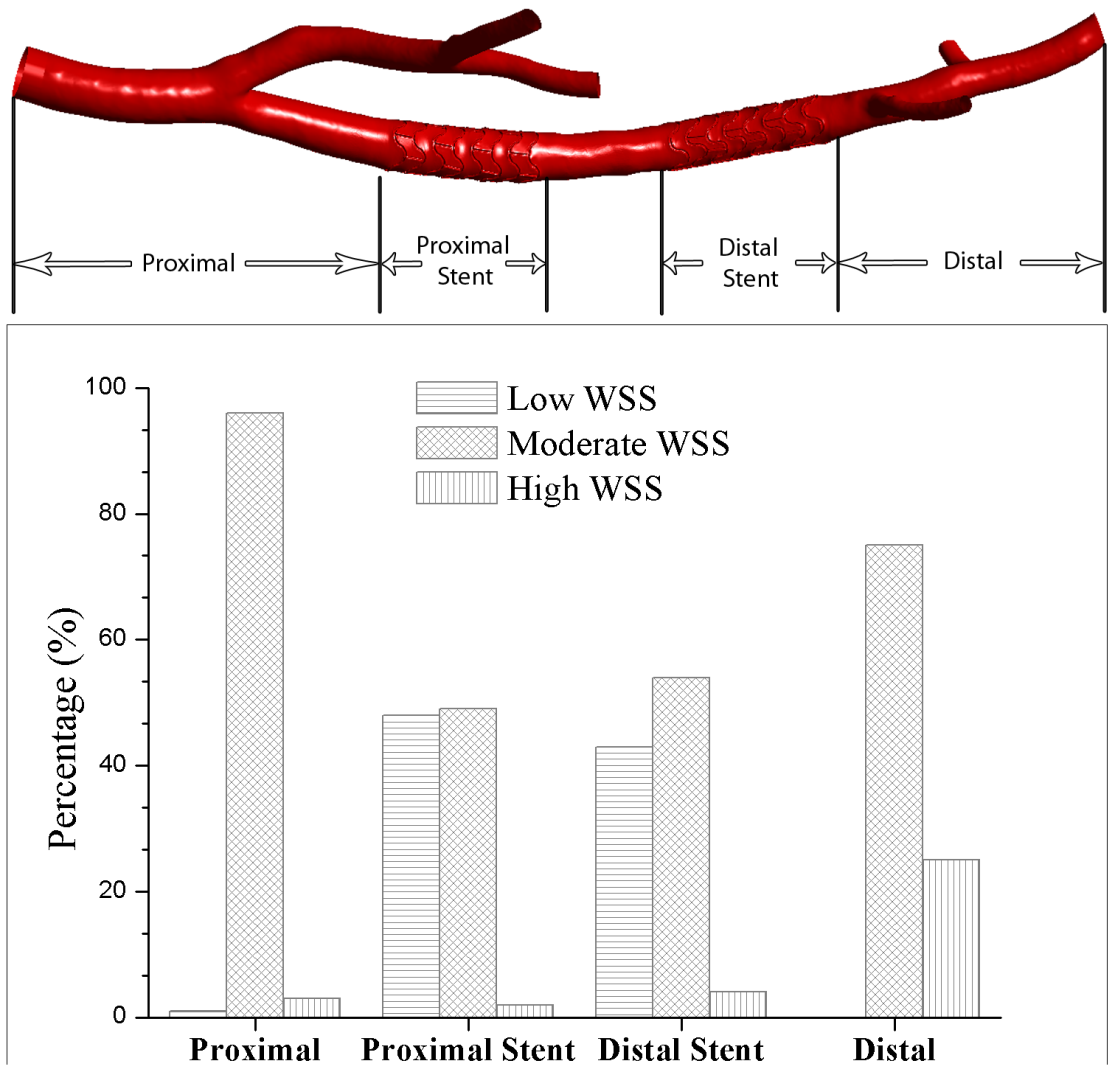
Distribution of relative vessel wall area exposed to different levels of shear stress. Results are shown in the bottom panel as percentage of the respective segment’s surface area in a porcine left coronary artery with two stents. Low wall shear stress: <0.5 Pa. Moderate: 0.5<WSS<2.5). High: >2.5 Pa. *Top:* Corresponding reconstructed surface of the arterial lumen negative.

The combined effect of local flow disturbance by the stent struts, changes in cross-sectional area and flow deflection as a result of axial arterial deformation is quantified in Fig. 9. There the distribution of low (<0.5 Pa), intermediate (0.5 to 2.5 Pa) and high (>2.5 Pa) WSS [11], [56], [57] is shown relative to the surface area of selected arterial sections in a vessel with two stents. These sections correspond to the area immediately upstream of the first stent (labeled ‘proximal’ in the top panel), the first and second stent (‘proximal stent’, ‘distal stent’), and the region after the second stent (‘distal’). In the stented sections, more than 40% of the wall surface area is exposed to low shear stress. In comparison, virtually no low WSS is present in the sections upstream of the first stent and downstream of the second stent. The high WSS observed after the second stent is due to the narrowing of the artery in that area.

## Discussion

As a result of the small feature sizes of stents and limited resolution of clinical imaging modalities, alternative methods have to be used to obtain the lumen geometry of stented arteries for calculation of WSS. It is accepted that wall shear stress affects vascular biology, influencing atherosclerotic plaque development and NIH. Here we have presented a method with which WSS can be determined by combining VCC, µCT and CFD. This method can be used in sacrificed animals or post mortem in humans after stenting has been performed either in vivo or ex vivo.

While similar approaches have been used before, this compound method removes some of the prior limitations: LaDisa et al. used VCC and µCT [43] with subsequent CFD modeling, but could not resolve individual stent struts. Morlacchi and co-workers stented pigs in vivo, excised the stented artery segments, embedded these in resin, acquired the deployed stent geometry with µCT and, in addition, performed histological analysis [32]. However, they could not acquire the lumen geometry, which had to be approximated for subsequent CFD analysis.

Since the presented method allows for processing stents deployed in vivo, it is expected to yield more accurate reconstruction of the in vivo WSS distribution than other methods that rely on µCT, but do not allow for in vivo stent deployment. Benndorf and co-workers observed marked differences between the WSS field obtained in a stented PTFE tube and an ex vivo stented preserved arterial segment [33]. This indicates that the choice of arterial wall representation can influence the derived hemodynamic parameters substantially. While one should expect that the preserved arterial segment mimics in vivo conditions better than the PTFE tube, the segment still showed artifacts in the form of circumferential creasing. Such creases are not observed in vivo and are presumably a result of the preservation process.

For comparison with virtual stent placement approaches, one has to consider whether these are capable of reproducing critical features observed in vivo. Our results show that neglecting tissue prolapse or stent malapposition may alter the reconstructed WSS field substantially. This is in accordance with the findings of Benndorf [33]. We further show that neglecting radial or axial arterial deformation will change WSS distribution as observed before by LaDisa [25], [26] and Murphy [46]. Virtual stent placement methods that cannot reproduce these critical features may have cost and processing time advantages, but are limited in the accuracy of WSS reconstruction. Assessment of virtual methods that do take into account critical features is less trivial. On the one hand, these methods are the first choice for predicting stent deployment and WSS distribution in live humans or animals. On the other hand, they idealize arterial wall mechanics, which is especially then critical when diseased arteries are investigated, as the presence of plaques and calcifications may change the wall properties substantially in an anisotropic and heterogeneous manner. Since the method introduced here can rely on the true geometry of vessels stented in vivo, we expect it to yield more accurate WSS readings than approaches based on virtual stent deployment. Of course, a suitable study is necessary to confirm this.

The presented method is based on three consecutively applied tools. These can each be optimized, but the interface between the methods has to be considered in the process. The optimal VCC resin has no shrinkage during curing and high stiffness thereafter. Biodur E 20 used here is a low shrinkage resin that produces rigid casts, maintaining the 3D configuration of the artery during maceration. The relative deviation of the cast lumen volume from the original one is less than 2%. There are other resins such as PU4ii that show lower shrinkage, but remain pliable after curing [58].

The resin should ideally have an X-ray attenuation behavior comparable to that of the stent to allow for optimal µCT imaging. Most VCC resins, however, have a very low attenuation coefficient, leading to low signal-to-noise ratio [59]. To increase resin opacity, we saturated the resin solvent, methyl ethyl ketone, with iodine [44]. This decreases viscosity, prolonging resin hardening time, and also increases shrinkage slightly. Alternatively, take-up of an attenuating compound after resin curing could be used, e.g. by bathing the cast in an aqueous solution of osmium tetroxide [59], [60]. However, this process is time consuming and only yields low penetration depths. In addition, OsO_4_ is very toxic.

The choice of scanner and acquisition settings has a great influence on the final results. The small size of the stent struts necessitates a high scan resolution, which in most scanners excludes the use of larger samples such as complete coronary artery tree casts. To circumvent this problem, we acquired the overall geometry and the stented section with two different scanners, introducing a time consuming registration step to merge the data. While a single step acquisition at high resolution would allow for a more automated work flow, it would result in very large datasets of over 30 gigabytes that are difficult to handle. In addition, scan time would increase by at least a factor of four, and scan cost would go up accordingly.

The quality of artery lumen reconstruction is the main determining factor for the accuracy of the WSS distribution calculations. Of similarly high importance is the choice of boundary conditions. Here we used a generic volumetric inflow rate or temporal profile, one zero pressure outlet and Murray’s law to set flow rates at the remaining outlets [47]. More accurate results could be obtained by applying a subject-specific inflow rate, which requires either phase contrast magnetic resonance imaging or invasive intravascular Doppler ultrasound measurements in vivo [21], [61], [62]. Boundary conditions at the outlets would ideally be determined by in vivo pressure or flow measurements as well. However, measurements in the distal artery segments are less accurate than in the larger parent vessels. Also, with increasing number of outlets, this approach becomes impractical. Applying Murray’s law, empirical variations thereof [63] or lower order models of the downstream vasculature to determine the boundary conditions at the outlets appear reasonable [29], [64].

Flow disturbances introduced by the stent lead to areas of low shear rate where blood displays non-Newtonian behavior. Consequently, a shear rate dependent rheology model should be used for best results. However, it is difficult to predict and to test which of the many existing models will give the most accurate results in stented arteries [23].

The main limitation of the presented method is that it entails destructive procedures. It can thus not be applied to live humans or to animals that should be kept alive. However, this does not exclude stenting in vivo and further processing ex vivo. When the method is applied to live animals, these can be treated without modifications to common protocols up to the point of sacrifice, after which VCC is started. If no in vivo acquisition of blood flow rates is foreseen in the original protocol, it should be added to derive realistic boundary conditions.

The method can also be used for post-mortem investigation of stented, atherosclerotic arteries in humans for research purposes. Processing of diseased vessels may require adaptation of individual process parameters. In particular, the possible entrapment of plaques and thrombi in the VCC resin and the presence of calcifications may render the image segmentation process more challenging [65], requiring changes in the concentration of the contrast agent and modification of µCT parameters. In addition, resin viscosity may need to be reduced if high grade stenoses are present [66]. Further studies are thus required to validate the performance of the method in diseased vessels.

Again due to the destructive nature of the method, histologic analysis and concurrent WSS derivation on the same artery are not possible. Consequently, a larger sample size is needed to statistically correlate vascular biology with WSS or other hemodynamic parameters. This adds to the comparably high cost of the method which derives from the large number of steps involved that each requires a high level of expertise. Next to in vivo stenting, the main cost factors are the high resolution µCT imaging and processing of the therewith associated large datasets.

Finally, unwanted interaction between the resin components and the stent may occur. In the current study such interaction resulted in gas bubbles, which were dealt with in the image segmentation process. It cannot be excluded that with other stents resin interaction may become a limitation.

In conclusion, the method presented herein constitutes a unique tool for accessing WSS in stented arteries. It can be employed to study the effects of hemodynamics on vascular biology, to develop stenting strategies that optimize hemodynamics and to design new stents that minimize regions of NIH promoting WSS.

## Supporting Information

Figure S1 **Cross-sections of stent struts acquired at (A) 6 µm, (B) 8 µm, (C) 10 µm and (D) 12 µm scan resolution.** Using the manufacturer’s production specifications as reference, the 6 µm resolution scan was found to capture the stent geometry with sufficient accuracy.
(TIF)

Figure S2 **Registration of the high resolution surface of a stented artery section with the lower resolution surface of the whole arterial geometry.** (A) Low resolution surface of the whole artery (B) High resolution surface of the stented region (C) Combined surface.
(TIF)

Figure S3 **Tetrahedral mesh cross-section at a stented artery section.** The inset shows the refined computational grid at the artery wall.
(TIF)

Figure S4 **Illustration of the bifurcation mass-flow conditions based on Murray’s law as given by Equations S1 and S2.**
(TIF)

Appendix S1 **Expanded Methods.**
(DOCX)

Video S1 **Reconstruction of stented LCA section and visualization of blood flow field.**
(MPG)

Video S2 **Visualization of flow disturbance introduced by malapposed stent strut.**
(MPG)
