# Supplementary figures and images for: Compound Ex Vivo and In Silico Method for Hemodynamic Analysis of Stented Arteries
Source: PLoS One. 2013 Mar 13;8(3):e58147. doi: 10.1371/journal.pone.0058147 (PMC3596389; doi:10.1371/journal.pone.0058147)

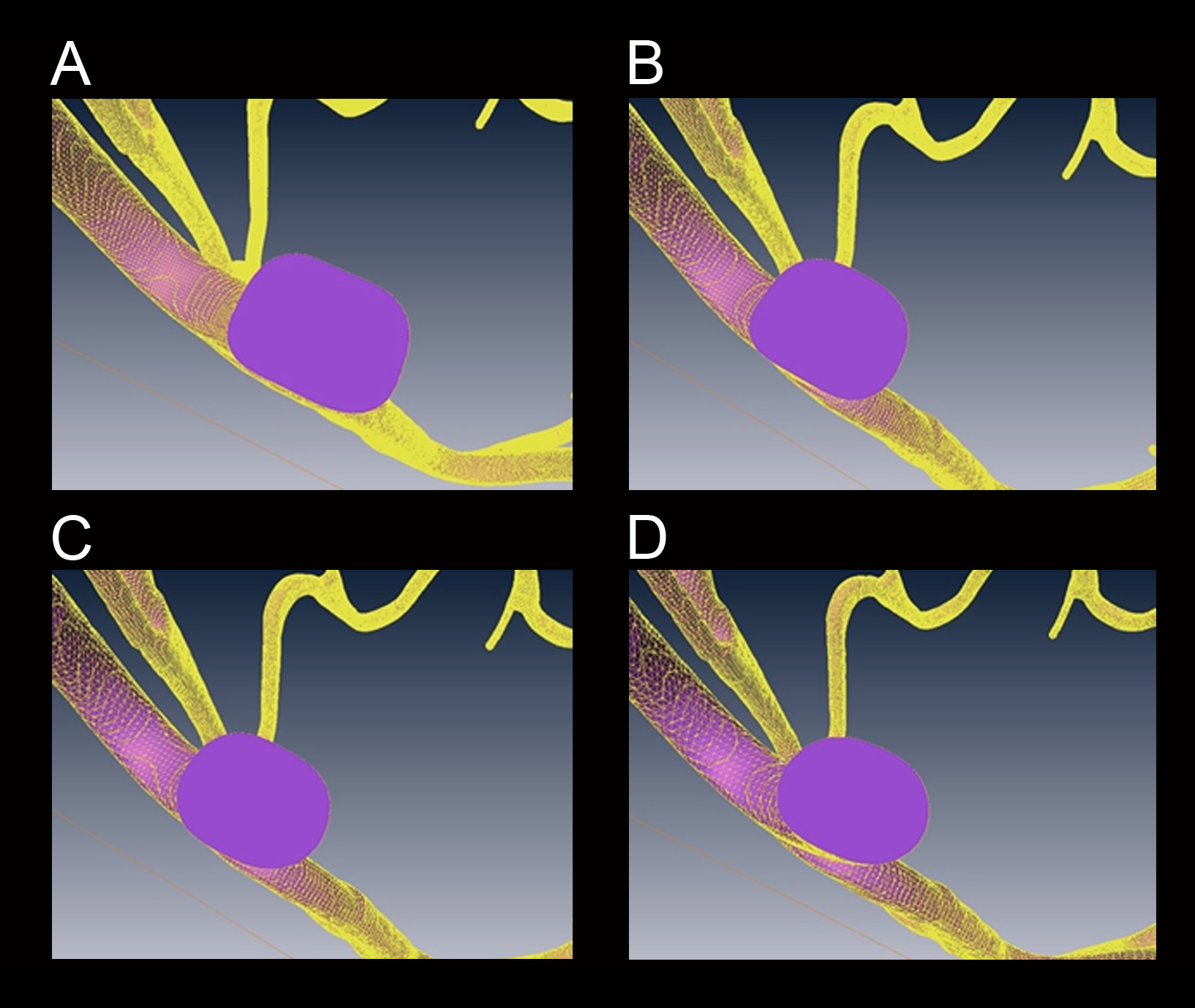

Supplement: Figure S1 — Cross-sections of stent struts acquired at (A) 6 µm, (B) 8 µm, (C) 10 µm and (D) 12 µm scan resolution. Using the manufacturer’s production specifications as reference, the 6 µm resolution scan was found to capture the stent geometry with sufficient accuracy. (TIF) [file pone.0058147.s001.tif]

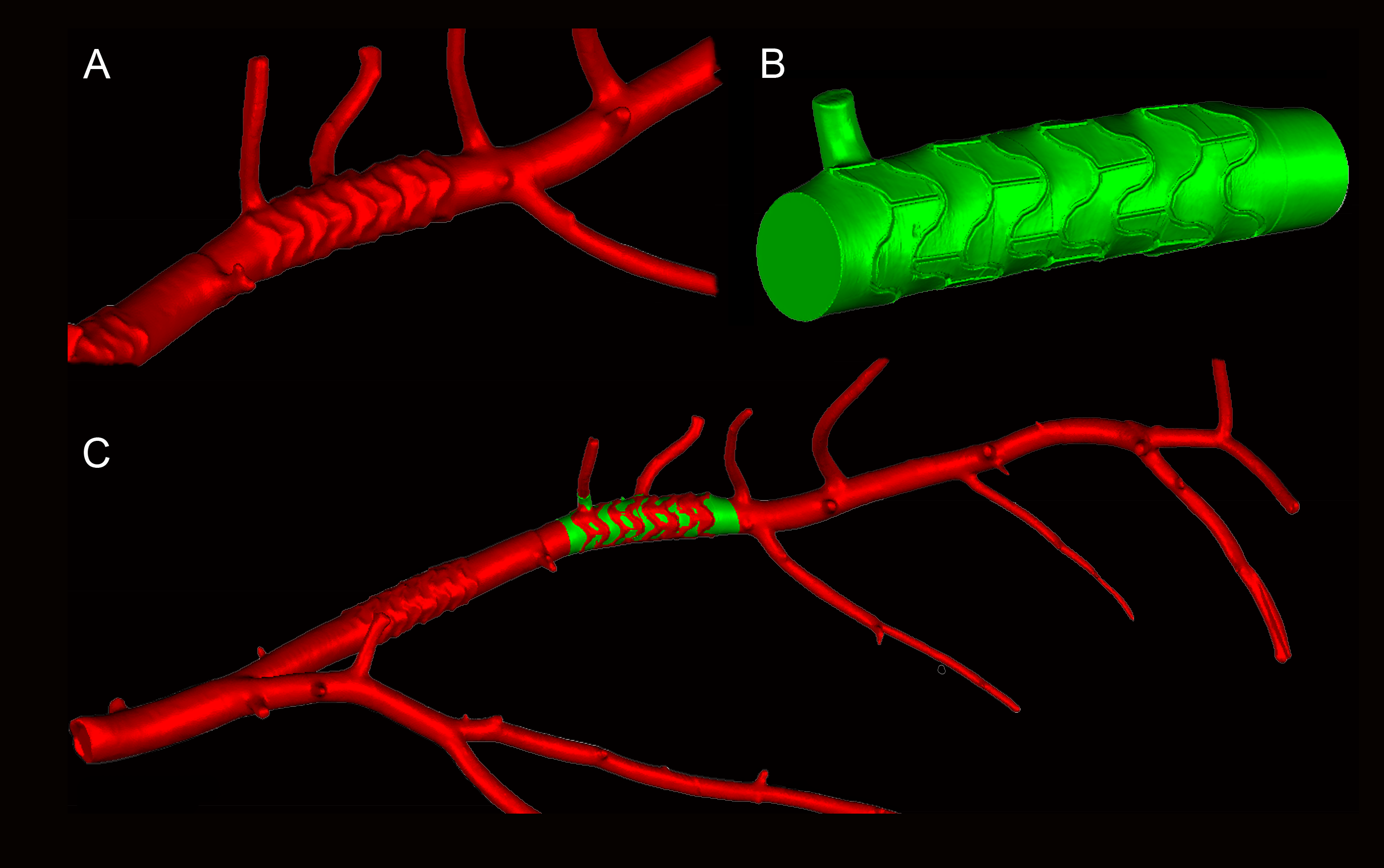

Supplement: Figure S2 — Registration of the high resolution surface of a stented artery section with the lower resolution surface of the whole arterial geometry. (A) Low resolution surface of the whole artery (B) High resolution surface of the stented region (C) Combined surface. (TIF) [file pone.0058147.s002.tif]

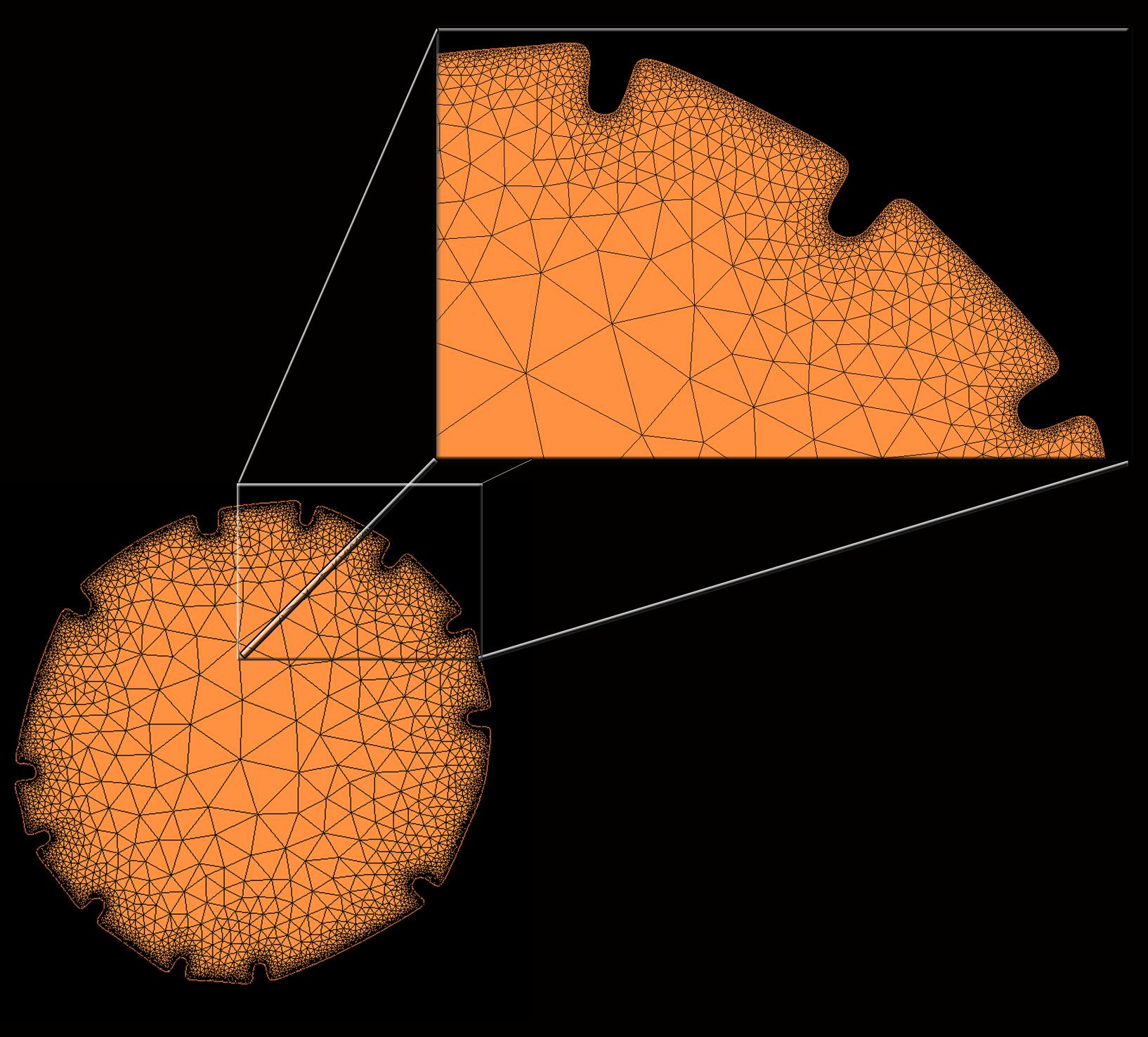

Supplement: Figure S3 — Tetrahedral mesh cross-section at a stented artery section. The inset shows the refined computational grid at the artery wall. (TIF) [file pone.0058147.s003.tif]

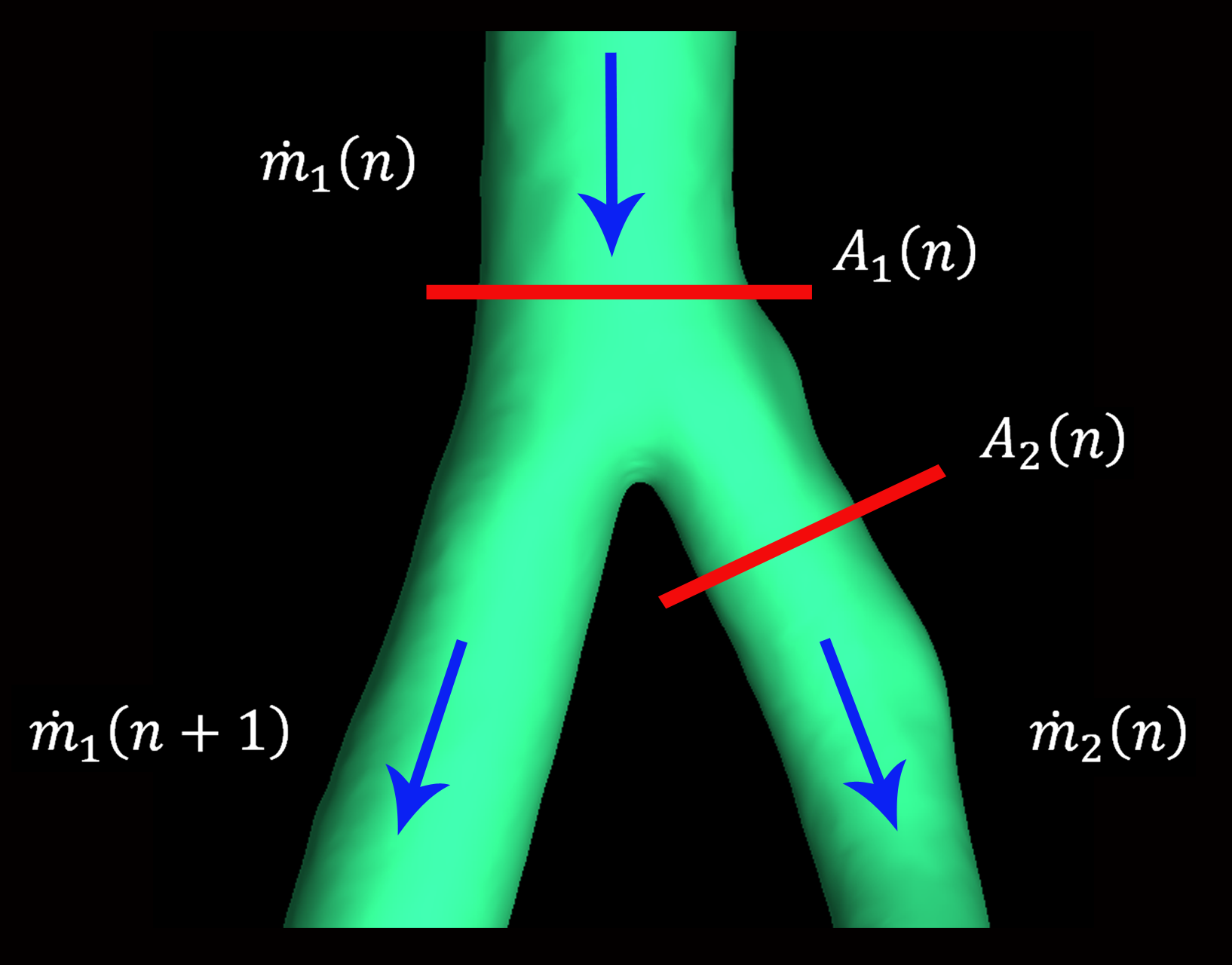

Supplement: Figure S4 — Illustration of the bifurcation mass-flow conditions based on Murray’s law as given by Equations S1 and S2. (TIF) [file pone.0058147.s004.tif]
