# Supplementary material for: Compound Ex Vivo and In Silico Method for Hemodynamic Analysis of Stented Arteries
Source: PLoS One. 2013 Mar 13;8(3):e58147. doi: 10.1371/journal.pone.0058147 (PMC3596389; doi:10.1371/journal.pone.0058147)
Supplement: Appendix S1 — Expanded Methods. (DOCX) [file pone.0058147.s005.docx]

# Appendix S1

## Expanded Methods

### Heart preparation and stenting

Fresh cadaveric porcine hearts with intact ascending aorta procured from the local slaughter house were used. All but the initial 10 mm of the aorta from the aortic valve was removed, and the heart was washed and weighed. The left coronary ostium was exposed, and a shortened 5 F sheath introducer cannula (Cordis Corporation AVANTI®) was inserted until its tip was located 25 mm distal to the ostium. The shortening was performed to reduce the pressure drop during resin injection for corrosion casting. The cannula was fixed using surgical ligation suturing in two places (approximately 1 and 2 cm distal to ostium) without puncturing the coronary artery. The artery was then flushed with a heparin solution and the heart was stored in saline solution and refrigerated. Within 24 hours, the hearts were removed from the refrigerator and were allowed to reach room temperature prior to stenting. A trained interventional cardiologist performed the stent placement under angiographic guidance. Biotronik absorbable metal scaffolds of 10 mm length and 3 mm diameter already mounted on a balloon catheter (Biotronik AG, Bülach, Switzerland) were deployed with 12 bar inflation pressure according to the manufacturer’s specifications.

### Vascular corrosion casting

Biodur E20 (EP20 - EP22), a low-shrinkage epoxy-based resin, was used for casting (Biodur Products GmbH, Heidelberg, Germany). Methyl ethyl ketone (MEK) was added to the resin to decrease viscosity and increase setting time, allowing for easier manipulation. Iodine already dissolved in MEK was added to the resin and hardener mixture at a ratio of 1:0.1225 (weight) to render it radio opaque. This ratio corresponds to 5% of iodine by mass in the final, dried resin. After mixing, the resin was degassed in a vacuum chamber for 2-3 minutes to remove any trapped air bubbles. Prior to resin injection, the heart was placed in a water bath and its left coronary artery (LCA) was flushed with water to remove air bubbles.

The resin injection was performed with a pressurized air system with controlled pressure to avoid over-inflation of the artery [1]. During most of the cardiac cycle, the coronary artery inlet pressure is equivalent to the aortic diastolic pressure. Therefore, a physiological pressure of 90 mmHg was used for the resin injection.

After injection, the cannula was closed using a built-in stopper while maintaining air pressure to allow the resin to set at a pressurized state. The heart was left submerged in water at room temperature for at least 36 hours while the resin hardened completely. Finally, the heart tissue was macerated at 55°C for 12 h using a 7.5% w/v solution of potassium hydroxide (KOH).

After maceration, the KOH solution was disposed of and the cast was washed in water with detergent to remove any remaining tissue or fat. Once dry, any small vessels (approximately <10% the size of the main branch cross-sectional area) were removed. This is acceptable, since such small vessels have limited effect on the hemodynamics in the main vessel branches [2‒4].

### Micro-computed tomography imaging of stented casts

The stented coronary artery cast was imaged using micro-computed tomography (μCT) in two steps: First, the entire cast was acquired at a resolution of 74 μm, and then the stented segment was scanned with 6 μm resolution. This was done to reduce scan time and limit data size while still fully resolving all geometric features that can influence hemodynamics Figure S1 visualizes the effect of scan resolution on acquired stent strut fidelity. Panels A to D show, respectively, the representations of strut cross-section at 6 µm, 8 µm, 10 µm and 12 µm scan resolution.

The lower resolution scans of the overall coronary artery were obtained using μCT (μCT80, Scanco Medical, Brüttisellen, Switzerland) with an isotropic voxel size of 74 μm (energy 70kVp, integration time 300 ms, tube current 114 μA, and two times frame averaging). The stented section of the artery was then cut and scanned at an isotropic voxel size of 6 μm (μCT40, energy 70kVp, integration time 300 ms, tube current 114 μA, and two times frame averaging). A constrained three-dimensional (3D) Gauss filter with a filter width of 1.2 and a support of 1 voxel was used to partly suppress noise in the raw μCT volumes. Finally, the data were converted to 2D image stacks in TIFF format for further processing.

### Reconstruction of 3D geometry

The high resolution tomographic image stack was segmented semi-automatically by thresholding with the image processing software Avizo (Visualization Sciences Group SAS, Merignac, France). Calibration of the segmentation was carried out via width comparison of the segmented inter-strut connectors to the manufacturer specified value of 70 µm.

The lower resolution image stack was also segmented in Avizo by thresholding. Calibration was carried out by comparing the segmented vessel diameter immediately proximal to the stent with the same diameter obtained via segmentation of the high resolution images. The surfaces of both segmented geometries were then triangulated and exported in STL format for further processing with Geomagic Studio 12 (Geomagic Inc., Morrisville, NC, USA), where the two geometries were registered automatically following an initial approximate alignment (Figure S2).

After registration, the surfaces were saved separately for individual processing, which was performed to remove any scanning artifacts and smooth the surfaces for a more efficient meshing. Once processed, the surfaces were merged in Geomagic by removing the stented section from the lower resolution dataset and then combining the remaining low and high resolution surfaces.

### Computational grid generation

An octree method in ANSYS ICEM CFD (ANSYS, Inc., Canonsburg, PA, USA) was used for the generation of a tetrahedral computational grid in the fluid domain. The precise calculation of wall shear stress (WSS) requires high spatial resolution at the domain walls. To ensure this, the mesh was refined at the artery walls and gradually coarsened towards the lumen center to keep computational cost at bay. Figure S3 shows a representative cross-section of a stented artery segment.

Independence of the computational results from the spatial discretization was tested via successive refinement of a nominal stented arterial tree and comparison of WSS obtained at each refinement step. A nominal grid density of 48 million tetrahedral elements per LCA was chosen.

### Boundary conditions

The coronary ostium, artery wall and distal ends of the coronary artery branches require the assignment of boundary conditions. No slip (zero velocity) was prescribed at the artery walls. Mass flow rate at the ostium based on literature values and mass flow rate ratios between branches were prescribed at the branch ends based on Murray’s Law, which derives from the minimization of energy required to pump and maintain blood [5]. Briefly, mass flow rate through a daughter branch in a bifurcation as shown in
Figure S4 is calculated using Equation S1.

 (S1)

where $\dot{m}$and A are mass flow rate and cross-sectional area, and the subscripts 1 and 2 correspond to the main and the first daughter branch, respectively.

To ensure mass conservation, the mass flow into a further daughter branch (n+1) is determined recursively based on the mass-flow leaving the previously calculated branches, i.e.

 (S2)

In case of bifurcations on daughter branches, area-weighted mass flow rate is specified at each outlet. Finally, the main branch outlet is set to zero pressure.

### Flow computations

The continuity and Navier-Stokes equations,

$\nabla. u = 0$ (S3)

$\rho\frac{\partial u}{\partial t} +\rho u . \nabla u = - \nabla p + \mu\nabla^{2} u$ (S4)

respectively, were solved numerically in ANSYS CFX (ANSYS Inc., Canonsburg, PA, USA) to obtain the velocity and pressure fields in the LCA. Here, u is velocity, t is time, ρ is density and p and μ are pressure and dynamic viscosity, respectively.

Assuming a Newtonian fluid model, WSS can be derived as the product of the dynamic viscosity and the shear strain rate, which is the derivative of the velocity in normal direction to the wall. Values of
1050 kg/m^3^ and 0.0035 Pa^.^s were used for density and viscosity, respectively. Residual reduction to 10^-8^ of the initial value was used as convergence criterion for the steady-state simulations, 10^-6^ for the transient ones. Oscillatory shear index (OSI) is evaluated for transient simulations according to Equation S5.

 (S5)

### Expanded Results

The supporting Video S1 shows the reconstruction of a stented left coronary artery section, and visualizes the blood flow field therein. In Video S2, the flow disturbance caused by a malapposed stent strut is shown.

## Supporting References

1. Griffiths DJ. (1998) Inject vessels with epoxy using compressed air and a simple jig. Journal of the International Society of Plastination 13: 15-16.

2. Zhao SZ, Xu XY, Collins MW, Stanton AV, Hughes AD, Thom SA. (1999) Flow in carotid bifurcations: effect of the superior thyroid artery. Medical Engineering & Physics 21: 207-214.

3. Myers JG, Moore JA, Ojha M, Johnston KW, Ethier CR. (2001) Factors influencing blood flow patterns in the human right coronary artery. Annals of Biomedical Engineering 29: 109-120 .

4. Steinman DA. (2002) Image-based computational fluid dynamics modeling in realistic arterial geometries. Annals of Biomedical Engineering 30: 483-497.

5. Murray CD. (1926) The physiological principle of minimum work: I. The vascular system and the cost of blood volume. Proceedings of the National Academy of Sciences of the United States of America 12: 207-214.
